# Common cuckoos (*Cuculus canorus*) affect the bacterial diversity of the eggshells of their great reed warbler (*Acrocephalus arundinaceus*) hosts

**DOI:** 10.1371/journal.pone.0191364

**Published:** 2018-01-19

**Authors:** Nikoletta Geltsch, Zoltán Elek, László Manczinger, Csaba Vágvölgyi, Csaba Moskát

**Affiliations:** 1 MTA-ELTE-MTM Ecology Research Group, a joint research group of the Hungarian Academy of Sciences, the Biological Institute of the Eötvös Loránd University and the Hungarian Natural History Museum, Budapest, Hungary; 2 Department of Ecology, Faculty of Science and Informatics, University of Szeged, Szeged, Hungary; 3 Department of Microbiology, Faculty of Science and Informatics, University of Szeged, Szeged, Hungary; Estacion Experimental de Zonas Aridas, SPAIN

## Abstract

The common cuckoo (*Cuculus canorus*) is an avian brood parasite, laying its eggs in the nests of other bird species, where these hosts incubate the parasitic eggs, feed and rear the nestlings. The appearance of a cuckoo egg in a host nest may change the bacterial community in the nest. This may have consequences on the hatchability of host eggs, even when hosts reject the parasitic egg, typically within six days after parasitism. The present study revealed the bacterial community of cuckoo eggshells and those of the great reed warbler (*Acrocephalus arundinaceus*), one of the main hosts of cuckoos. We compared host eggs from non-parasitized clutches, as well as host and cuckoo eggs from parasitized clutches. As incubation may change bacterial assemblages on eggshells, we compared these egg types in two stages: the egg-laying stage, when incubation has not been started, and the mid-incubation stage (ca. on days 5–7 in incubation), where heat from the incubating female dries eggshells. Our results obtained by the 16S rRNA gene sequencing technique showed that fresh host and cuckoo eggs had partially different bacterial communities, but they became more similar during incubation in parasitized nests. Cluster analysis revealed that fresh cuckoo eggs and incubated host eggs in unparasitized nests (where no cuckoo effect could have happened) were the most dissimilar from the other groups of eggs. Cuckoo eggs did not reduce the hatchability of great reed warbler eggs. Our results on the cuckoo-great reed warbler relationship supported the idea that brood parasites may change bacterial microbiota in the host nest. Further studies should reveal how bacterial communities of cuckoo eggshells may vary by host-specific races (gentes) of cuckoos.

## Introduction

Studies on the diversity of microbiota, including bacteria, receive considerable attention by animal ecologists, as bacteria are fundamental components of animal bodies. They live on skin, scales, feathers, fur and exoskeletons, in digestive, respiratory and reproductive tracts, and in specialized glands for grooming, preening or olfactory communication [1]. In avian ecology studies have begun to describe the role of avian microbiota in bird nests. For example, bacteria could be transmitted from nest material to the eggshell, as it was revealed in reed warblers (*Acrocephalus scirpaceus*) [2] and hoopoes (*Upupa epops*) [3]. Nest visitors, such as brood parasites, may affect the microbiome of the nests and thus change their bacterial environment [4]. Consequently, they might selectively influence the viability of embryos [4, 5]. Several behaviours, such as blood-sucking and defecation by ectoparasites [6] or damaging of eggs by brood parasites [7], may also affect the microbiome of avian nests by diversifying or partially exchanging their bacterial community.

A previous study on brood parasitic great spotted cuckoos (*Clamator glandarius*) revealed that these brood parasites visit the nests of their Eurasian magpie (*Pica pica*) hosts several times, and may damage one or more host eggs. This behaviour may increase the risk of bacterial contamination [4]. Consequently, higher bacterial loads were found in parasitized than in non-parasitized nests. Moreover, the bacterial load was lower on the surface of great spotted cuckoo eggs than on host eggs [4]. They suggested that the parasitic eggs are adapted better to the environment than those of their magpie hosts because of the higher risk of bacterial contamination, e.g., damaged host eggs. The effects of brood parasitisms on hygiene, i.e. the bacterial environment of nests, may increase the costs of parasitism from the viewpoint of hosts in brood parasitic relationships. This study and several other previous studies on the bacteria of avian eggshells (e.g., [8, 9]) used general and specific agar media to identify bacterial isolates. For a more detailed estimation our study characterizes the microbial diversity of eggshells of common cuckoos (*Cuculus canorus*) and their great reed warbler (*Acrocephalus arundinaceus*) hosts through the 16S rRNA gene-based sequencing approach.

Avian incubation seems to be an effective tool for birds to protect their clutches from bacterial infection. For example, a previous study on the cavity nester tree swallow (*Tachycineta bicolor*) revealed that bacterial growth was accelerated when the incubation was inhibited and the eggs stayed wet. This study provided experimental evidence that keeping eggs dry can be regarded as the mechanism responsible for antimicrobial effects in avian incubation [8]. Another study by Shawkey et al. [10] revealed that incubation inhibits growth and diversification of bacterial communities on the eggshells of a box-nesting population of pearly-eyed thrashers (*Magarops fuscatus*). An analysis of pied flycatcher (*Ficedula hypoleuca*) eggs showed a connection between early incubation and an inhibition of bacterial proliferation through a drying effect on eggshells [9]. In the reed warbler incubation caused the extinction of potentially harmful Gram-negative bacteria on reed warbler eggshells [2]. Consequently, incubation intensity negatively affected eggshell bacterial diversity, while relative humidity positively associated with eggshell bacterial loads for heterotrophic bacteria, Gram-negative bacteria and the genus *Pseudomonas*, although the significance of these associations varied between bacterial groups [9].

The purpose of our study was to investigate the biodiversity of cultivable bacteria in the common cuckoo and one of its main host species [11], the great reed warbler. This cuckoo species is an obligate brood parasite laying their eggs in their hosts' nests, in our case in the nests of great reed warblers. Some of the cuckoo eggs are lost (ca. 33%) if great reed warblers recognize the parasitic eggs and reject them from the nests (within 6 days) by egg ejection, nest desertion or egg burial [12]. If the parasitic egg is accepted and incubated together with the host's own eggs, the early-hatching young cuckoo chick evicts all nest content within three days after hatching [13]. Consequently, it utilizes all parental care of their foster parents.

In the present study we compared the eggshell bacterial community of great reed warblers together with that of common cuckoos in central Hungary. Although common cuckoos do not break host eggs as it was reported from the Clamator system [4], bacterial contamination could also be expected even when laying does not necessarily imply host egg breakage. We hypothesized that brood parasitism increases the bacterial load on host eggshells in parasitized clutches. Consequently, we predicted higher bacterial loads on great reed warbler eggshells in parasitized than in non-parasitized nests. However, cuckoos are separated into host-specific races, the so-called gentes [14], and female cuckoos typically lay their eggs in the nests of the same host species that raised them. Consequently, we expected similar bacterial community on the eggshells of both the hosts and the corresponding brood parasite gentes as an alternative hypothesis. In this case we predicted similar loads in parasitized and non-parasitized nests in the great reed warbler—common cuckoo relationship. We compared eggshell bacterial community in two states of breeding, i.e. in the egg-laying stage ('non-incubated' eggs), and the incubation stage ('incubated' eggs). As incubation is supposed to reduce bacterial loads by the heat (drying) effect during incubation (see above), we consequently predicted more difference between the bacterial loads of host and parasitic eggs in the laying stage than during incubation.

## Materials and methods

### Study area and sampling

Field work was carried out about 40 km south of Budapest, Hungary, in the surroundings of Apaj (47°07’N, 19°05’E), between mid-May and mid-July in 2012. In our study area great reed warblers nest in reed (*Phragmithes australis*) beds that grow in 2–4 m wide strips along both sides of small flood relief and irrigation channels. The modal clutch size of great reed warblers is 5 eggs and modal brood size is 4 chicks at this site [15]. Only females incubate the eggs (ca. for 12 days in our study area), and incubation starts just after the fourth egg is laid, i.e. typically one day before clutch completion [16]. A high proportion (41–68%) of host nests is parasitized by cuckoos, representing an unusually high level of cuckoo parasitism [17].

We collected 71 samples from 47 nests, including parasitized and non-parasitized clutches (Table 1). Samples were taken in the field from eggshells, attempting to keep the conditions as aseptic as possible. New latex gloves sterilized with 96% ethanol were used for each nest to prevent inter-nest contamination. Once the gloves were dry, we gently handled and sampled eggs by rubbing the complete eggshell using the Whatman, Buffer Swab system. In this system each sterile swab is stored in individual tubes containing 2 ml of sterile phosphate saline buffer (PBS) (monopotassium phosphate 42.5 mg/l, potassium di-hydrogen phosphate 34 g/l,pH 7.2 ± 0.5). We randomly sampled one egg of the same species in each nest with a single swab. We collected samples from nests only once during the entire experiment, so non-incubated and incubated eggs were sampled in different nests. The complete egg surface was wiped. After taking samples the swabs were placed back to sterile tubes and transported in a portable refrigerator at 4–6°C. Six treatments were formed for collecting samples (Table 1). For the sake of simplicity we call these groups treatments, although no experiment was performed.

**Table 1:** Number of bacterial samples from cuckoo and great reed warbler eggshells. (Acronyms of categories used in the study: pcn = parasitized clutch, cuckoo egg, non-incubated; pci: parasitized clutch, cuckoo egg, incubated; pgn = parasitized clutch, great reed warbler egg, non-incubated; pgi = parasitized clutch, great reed warbler egg, incubated; ngn = non-parasitized clutch, great reed warbler egg, non-incubated; ngi = non-parasitized clutch, great reed warbler egg, incubated).

Species	Non-incubated eggs	Incubated eggs	Total
**P**arasitized nest			
**C**uckoo	10 (**pcn**)	14 (**pci**)	24
** G**reat reed warbler	10 (**pgn**)	14 (**pgi**)	24
**N**on-pararasitized nest			
** G**reat reed warbler	13 (**ngn**)	10 (**ngi**)	23

Bacteria from eggshells were sampled in non-parasitized and parasitized nests, in two stages: in the egg-laying stage (fresh eggs) and, in different nests, during incubation (incubated eggs). The first samples were taken 1–2 days before clutch completion (non-incubated eggs), and the second samples were collected during the incubation period (ca. on days 5–7 in incubation). Samples were carried in a cool-box and stored in a fridge both in the field station and in the lab (5°C). They were analysed within 5 days after collection, and all samples were treated blind.

### Isolation and cultivation of bacteria

Isolation of bacteria was performed by homogenously spreading 0.1 ml of the samples onto the surfaces of media in Petri dishes. Before culturing samples they were shaken in a vortex for at least three periods of 5 s. Four growth media (Biolab Inc., Budapest) were used: Vogel–Johnsson Agar (VJ) for *Staphylococcus*, Kenner Fecal Agar (KF) for *Enterococcus*, Hektoen Enteric Agar (HE) for *Enterobacteriaceae* and Tryptic Soy Agar (TSA) for heterotrophic bacteria to isolate diverse bacterial morphotypes (S1 Table). The plates were incubated aerobically at 37°C and colonies were counted 72 h after inoculation. There was no bacterial growth on Kenner Fecal Agar (KF) from any sample. This KF agar was eliminated from the subsequent analysis. Based on the colony morphology and pigmentation, distinct bacterial isolates were selected and subsequently isolated in pure cultures. With a view to compare the cultivable bacterial diversity among the samples, a wide range of bacterial isolates were studied. The isolates on master plates with Tryptic Soy Agar (TSA) were kept in refrigerator at 5°C until analysis.

### DNA extraction of isolates

Genomic DNA was isolated from bacterial cultures grown in 5 ml LB (Luria-Bertani liquid medium: 0.5% Yeast extract, 1% Tryptone, 1% NaCl) medium at 37°C overnight. Total genomic DNA was extracted by classical standard protocol. Two ml of sample was pipetted into microcentrifuge tube. After centrifugation of the sample at 3500 g for 5 min, supernatant was discarded. Cells were resuspended in 500 μl lysis buffer (1% SDS, 50 mM EDTA, 100mM TRIS pH = 8) and the tube vortexed for 3 min. After this, 275 μl of 7 M ammonium-acetate was added to the cells and the tube was incubated at 65°C for 5 min and kept on ice for 5 min. Chloroform-isoamylalcohol (24:1; 500 μl) was added to the mixture and centrifuged at 16,200 g for 10 min. The upper phase (approximately 500 μl) was transferred to a new microcentrifuge tube and 500 μl of isopropanol were added and kept on -20°C for 5–10 min. After centrifugation at 16,200 g for 10 min the supernatant was discarded and 500 μl of 70% ethanol was added to the pellet. After centrifugation at 16,200 g for 5 min the supernatant was discarded and the pellet was dried. Finally, the pellet was diluted in 30 μl bi-distilled water. The DNA quality was checked by agarose (1%, w/v) gel electrophoresis.

#### Polymerase chain reaction for 16S rRNA gene sequences

The 16S rRNA gene sequence was amplified using universal primers 27F (5′ GAGTTTGATCCTGGCTCAG 3′) and 1492R (5′ ACGGCTACCTTGTTACGACTT 3′) [9, 18]. The reaction mixture (20 μl) consisted of 0.5 U of DreamTaq Polymerase (Thermo Fisher, USA), 2 μl of 10 x DreamTaq buffer, 0.4 mM of dNTP mix, 10 pmol of each primer, and 1 μl of template DNA (~50 ng). The polymerase chain reaction (PCR) program was set as follows: initial denaturation at 95°C for 2 min, followed by 30 cycles for 30 sec at 94°C, 1 min at 60°C, and 1 min 10 sec at 72°C, and a final extension cycle at 72°C for 10 min.

### Phylogenetic analysis of 16S rRNA gene sequences

A total of 177 representative isolates were sequenced (S1 Table) and their PCR amplicons were purified by 1% agarose gel extraction kit (Qiagen, USA). DNA sequences were manually checked and carefully edited by MEGA 6.0 [19]. Sequences were aligned by MAFFT v7.244. Maximum Likelihood (ML) analysis was performed by using raxmlGUI v1.5b1 [20] under the GTR model with GAMMA-distributed rate heterogeneity with 1000 bootstrap replicates. Classification of the bacterial sequences was carried out by comparing them to those in the GenBank database, using the Basic Local Alignment Search Tool algorithm (BLAST) nt/nt [21].

### Statistical analysis

All analyses were performed in R 3.3.0 [22]. We ran hierarchical cluster analysis with the "hclust" function in the "stats" package. This method is a tool for exploring data structure (e.g. [23]) by grouping objects in a hierarchical way. In cluster analysis the procedure matrix was calculated by the Jaccard's index and clusters were amalgamated by the average method. We applied Linear Discriminant Analyses (LDA, [24]) to explore how cuckoo eggs may affect the bacterial community in the nests. This method calculates a linear combination of the predictors that gives maximum separation between the centers of the data while minimizing the variation within each group of data. We also used LDA for the classification of samples, as LDA is suitable to predict group membership in mutually exclusive groups [25]. We used a model formula where the clutches, as a basis of data classes, were compared by the matrix of bacterium species per eggshell samples. In order to test the robustness of LDA, we compared the observed vs. fitted classes of predictions in the MASS [25] package.

## Results

### Bacterial genera and species

**Fig 1:**
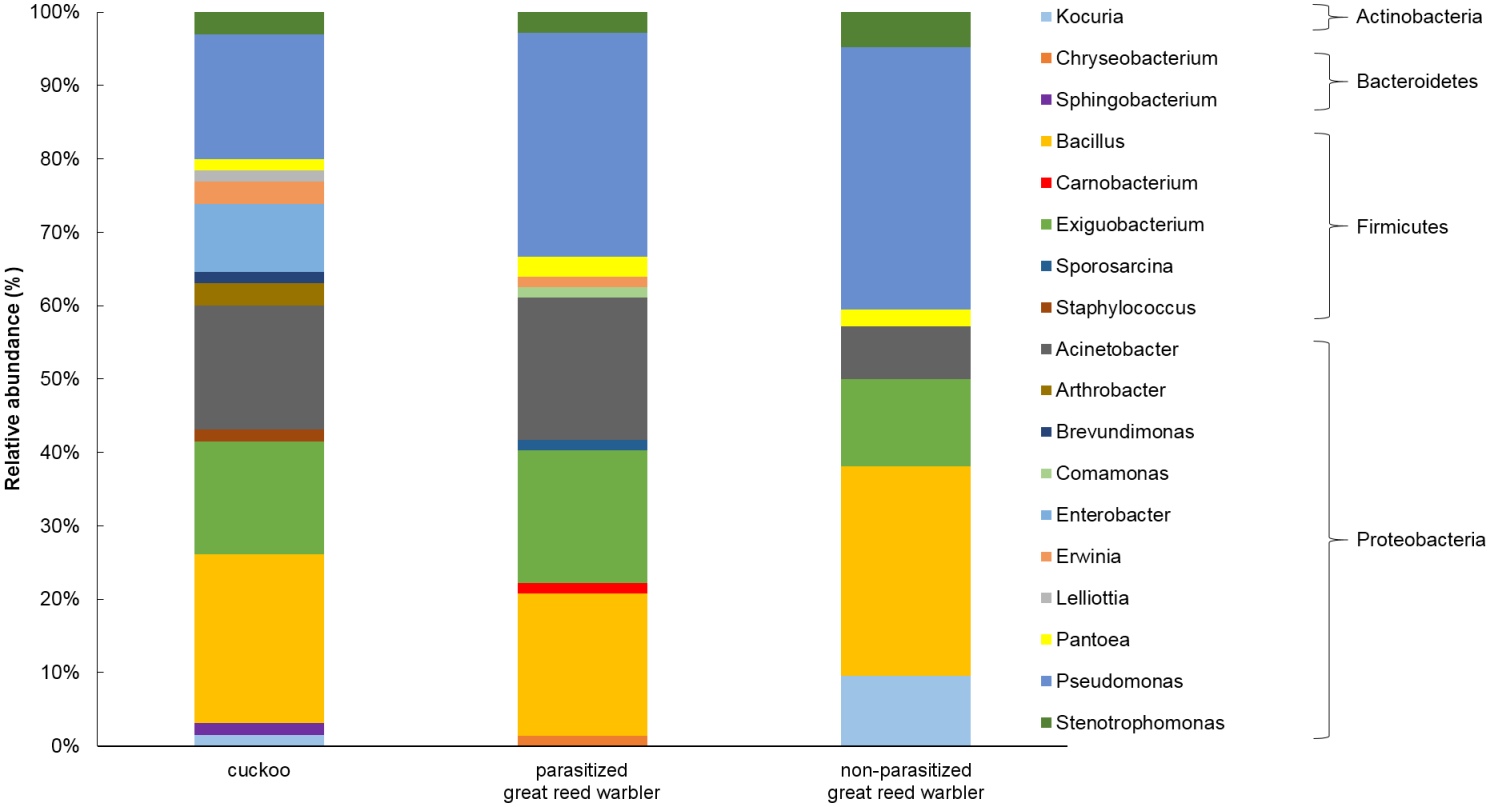
Relative abundance of bacterial genera on cuckoo and great reed warbler eggshells.

The study revealed 18 different genera (Table 2) which belonged to four phyla (Fig 1, S1 Fig), namely *Proteobacteria*, *Firmicutes*, *Actinobacteria* and *Bacteroidetes*. *Proteobacteria* (64.86%) and *Firmicutes* (27.03%) phyla were found to be predominant. A total of 177 bacterial isolates were obtained from the eggshell samples of cuckoos and great reed warblers (98 isolates were Gram-negative and 79 were Gram-positive, distributed in 11 and 7 genera, respectively).

**Table 2:** Number and percent (in brackets) of eggshells of cuckoos and great reed warblers with corresponding detected bacterial genera.

Bacterial genera	Parasitized nest						Non-parasitized nest		
	Cuckoo eggshell samples			Great reed warbler eggshell samples					
	Non-incubated	Incubated	Total	Non-incubated	Incubated	Total	Non-incubated	Incubated	Total
Gram-negative									
** ***Acinetobacter*	2 (11.1)	7 (15.6)	9 (14.3)	6 (22.2)	8 (17.8)	14 (19.4)	3 (8.8)	0	3 (7.1)
** ***Brevundimonas*	0	1 (2.2)	1 (1.6)	0	0	0	0	0	0
** ***Chryseobacterium*	0	0	0	1 (3.7)	0	1 (1.4)	0	0	0
** ***Comamonas*	0	0	0	0	1 (2.2)	1 (1.4)	0	0	0
** ***Enterobacter*	0	6 (13.3)	6 (9.5)	0	0	0	0	0	0
** ***Erwinia*	2 (11.1)	0	2 (3.2)	0	1 (2.2)	1 (1.4)	0	0	0
** ***Lelliottia*	0	1 (2.2)	1 (1.6)	0	0	0	0	0	0
** ***Pantoea*	1 (5.6)	0	1 (1.6)	0	2 (4.4)	2 (2.8)	1 (2.9)	0	1 (2.4)
** ***Pseudomonas*	4 (22.2)	7 (15.6)	11 (17.5)	7 (25.9)	15 (33.3)	22 (30.6)	11 (32.4)	4 (50)	15 (35.7)
** ***Sphingobacterium*	0	1 (2.2)	1 (1.6)	0	0	0	0	0	0
** ***Stenotrophomonas*	1 (5.6)	1 (2.2)	2 (3.2)	2 (7.41)	0	2 (2.8)	2 (5.9)	0	2 (4.8)
Gram-positive									
** ***Arthrobacter*	0	2 (4.4)	2 (3.2)	0	0	0	0	0	0
** ***Bacillus*	3 (16.7)	12 (26.7)	15 (23.8)	7 (25.9)	7 (15.6)	14 (19.4)	10 (29.4)	2 (25)	12 (28.6)
** ***Carnobacterium*	0	0	0	0	1 (2.2)	1 (1.4)	0	0	0
** ***Exiguobacterium*	4 (22.2)	6 (13.3)	10 (15.9)	4 (14.8)	9 (20.0)	13 (18.1)	3 (8.8)	2 (25)	5 (11.9)
** ***Kocuria*	0	1 (2.2)	1 (1.6)	0	0	0	4 (11.8)	0	4 (9.5)
** ***Sporosarcina*	0	0	0	0	1 (2.2)	1 (1.4)	0	0	0
** ***Staphylococcus*	1 (11.1)	0	1 (1.6)	0	0	0	0	0	0

We identified 12 genera and 25 species from great reed warbler eggshell samples and 14 genera and 27 species from cuckoo eggshell samples (S1 Table). From great reed warbler samples, the most frequent genera identified were *Pseudomonas* (32.46%), *Bacillus* (22.81%), *Exiguobacterium* (15.79%) and *Acinetobacter* (14.91%). These genera were also the most frequently isolated from cuckoo samples, but the order of their frequency was different. *Bacillus* (23.81%) was the most frequent genus on cuckoo eggshells, *Pseudomonas* was the second most common genus (17.46%) and *Exiguobacterium* approx. equally as frequent on cuckoo eggshell samples (15.87%) as great reed warbler eggshell samples (15.79%). Additionally, *Acinetobacter* was also frequent (14.29%). Other genera isolated with rates lower than 10% are shown in Table 2. *Brevundimonas* spp., *Enterobacter* spp., *Lelliottia* spp., *Sphingobacterium* spp. and *Staphylococcus* spp. were not isolated from the eggshells of any of the great reed warblers examined. *Carnobacterium* spp., *Chryseobacterium* spp., *Comamonas* spp. and *Sporosarcina* spp. were not isolated from the cuckoo eggshells. Genera that were more frequently isolated from great reed warblers than from cuckoos were *Pseudomonas* (32.46 vs 17.46%; p<0.05), *Kocuria* (3.51 vs 1.59%; p>0.05) and *Pantoea* (2.63 vs 1.59%; p>0.05). The genus *Enterobacter* was isolated from 9.52% of cuckoo eggshells, but was not isolated from great reed warbler eggshell samples.

Our results on particular bacteria species are as follows. *Acinetobacter johnsonii*, *Bacillus pumilus*, *Exiguobacterium undae* and *Pseudomonas putida* were identified from both non-incubated and incubated cuckoo and great reed warbler eggshells in parasitized nests (pcn, pci, pgn, pgi). *Acinetobacter lwoffii*, *Bacillusamyloliquefaciens*, *Bacillus subtilis* and *Pseudomonas orientalis* were found also on these eggshells, excet for the non-incubated cuckoo eggs. *Stenotrophomonas rhizophila* was on pcn, pci and pgn eggshells. We identified *Kocuria rhizophila* and *Sphingobacterium faecium* from incubated cuckoo eggshells. *Chryseobacterium indoltheticum* was just on non-incubated great reed warbler eggs in parasitized nests. *Enterobacter aerogenes* and *Enterobcter amnigenus* were only isolated from incubated cuckoo eggshells.

The Maximum Likelihood tree (S2 Fig) based on the 16S rRNA gene sequences showing the phylogenetic relationship of the cuckoo and great reed warbler bacterial isolates from parasitized nests.

### Hatchability of host eggs in parasitized clutches

We tested whether the presence of a cuckoo egg in a clutch affected hatchability of great reed warbler eggs in nests where the host eggs could be hatched before the young cuckoo chick evicted them. As the young cuckoo evicts all host eggs from the nest within three days after hatching [13, 26], most great reed warbler eggs have no chance of hatching, independently from bacterial infection, so we looked for cases in our database, when the host eggs hatched earlier. We compared the hatchability of great reed warbler eggs in parasitized and non-parasitized nests, measured as percent hatching of eggs, and found no difference (Mann-Whitney U-test_45,7_ = 107.5, P = 0.184).

### Statistical comparison of bacteriological samples

**Fig 2:**
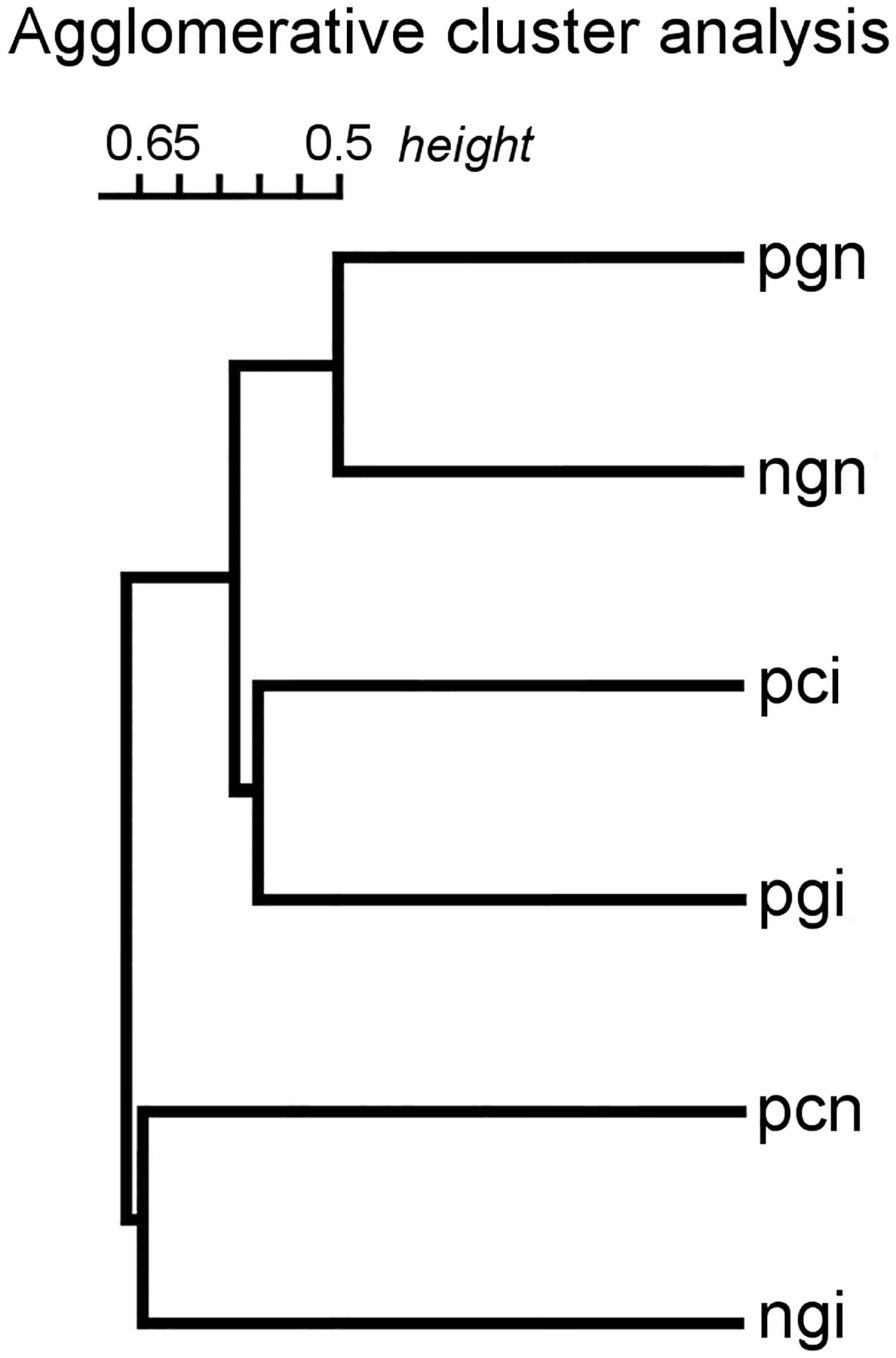
Dendrogram of agglomerative hierarchical cluster analysis. (Treatments: non-incubated clutches: ngn—non-parasitized great reed warbler egg, pgn—parasitized great reed warbler egg, pcn—parasitized cuckoo egg; incubated clutches: ngi—non-parasitized great reed warbler egg, pgi—parasitized great reed warbler egg, pci—parasitized cuckoo egg).

The dendrogram of hierarchical cluster analysis revealed the similarity structure of the bacterial communities (Fig 2). The non-incubated great reed warbler eggs in non-parasitized and parasitized nests (ngn and pgn) were clustered together, as host eggs could not be influenced by cuckoo eggs in unparasitized clutches and host eggs in parasitized nests in the egg laying stage was only a relatively short time (0–1 days) in contact with the cuckoo egg. However, incubated cuckoo and great reed warbler eggs (pci and pgi) were clustered together. We assume that the bacterial communities of cuckoo and great reed warbler eggs become similar during incubation. Following this logic, it is understandable that incubated great reed warbler eggs in non-parasitized nests and non-incubated cuckoo eggs (ngi and pcn) proved to be the most unique categories.

**Fig 3:**
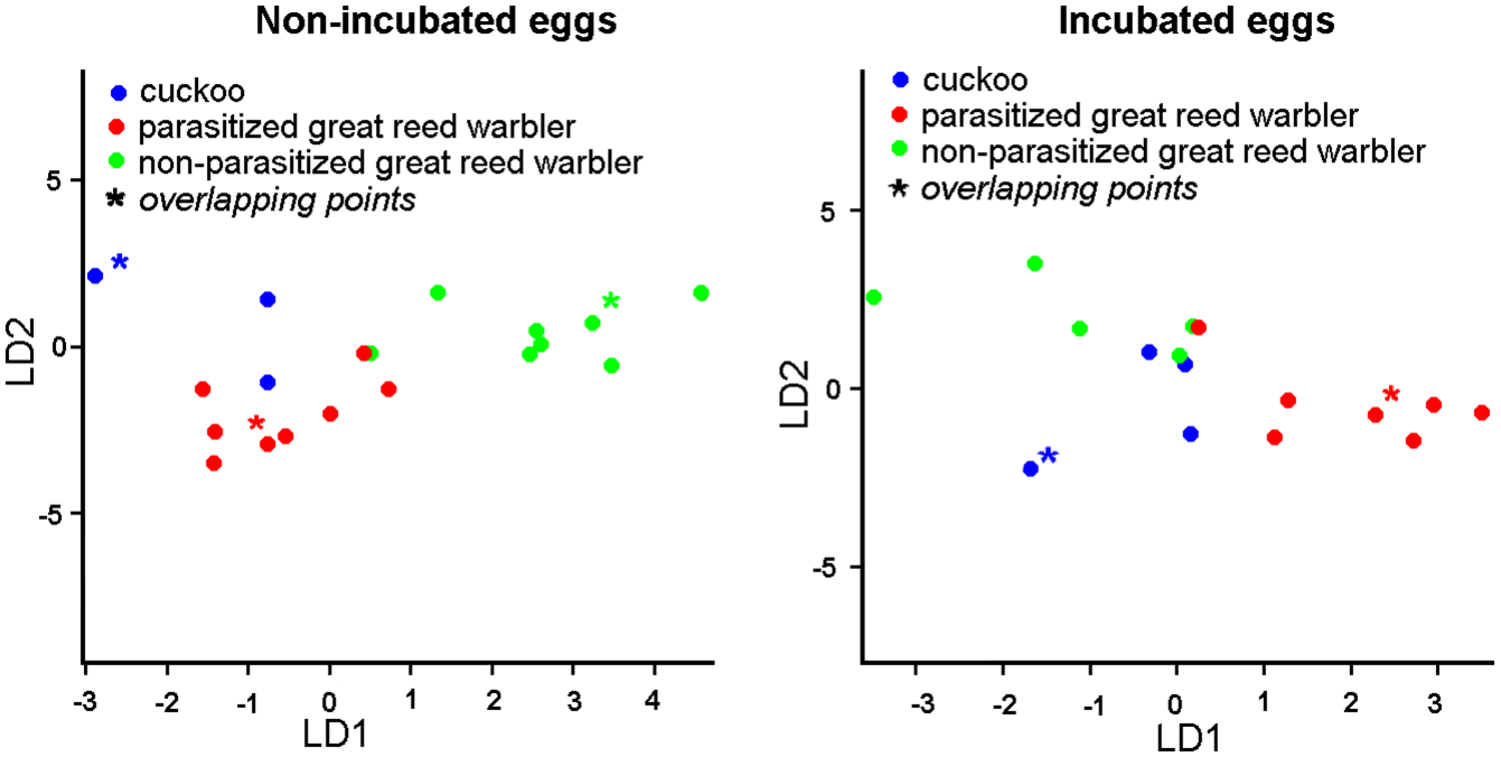
Plot of discriminant scores generated by Linear Discriminant Analysis, showing the bacterial community structure of cuckoo and great-reed warbler eggs.

Linear Discriminant Analysis plots were generated either for groups of non-incubated eggs (pcn, pgn, ngn) or incubated eggs (pci, pgi, ngi). These analyses revealed the fine-scale structure of bacterial samples regarding the highest potential for separation of the groups, as LDA ordinated individual samples into predefined two dimensions (coefficients of linear discriminants for non-incubated eggs: LD1: 0.63, LD2: 0.36; for incubated eggs: LD1: 0.67, LD2: 0.32; Fig 3). Both of our analyses revealed high accuracy of membership classification, i.e. the concordance between observed and predicted group memberships of individual samples (Table 3). This suggests the relative distinctness of groups and validated the usefulness of LDA in our case.

**Table 3:** Classification results of LDA for eggshell bacterial communities.

Treatment	Predicted group membership		
Non-incubated eggs	Cuckoo	Great reed warbler-1	Great reed warbler-2
Cuckoo	6	0	0
Great reed warbler-1 (in non-parasitized nest)	1	9	1
Great reed warbler-2 (in parasitized nest)	0	0	9
Incubated eggs	Cuckoo	Great reed warbler-1	Great reed warbler-2
Cuckoo	9	0	0
Great reed warbler-1 (in non-parasitized nest)	0	12	0
Great reed warbler-2 (in parasitized nest)	2	1	5

## Discussion

We revealed that cuckoo parasitism can be regarded as a bacterial vector effect in the nests of their hosts, as we found significant differences in the bacterial community in parasitized host nests when compared with non-parasitized host clutches. We characterised and compared the eggshell cultivable bacteria of these two avian species, and showed that cuckoos changed the hygienic conditions of host nests. Even though the analysis of microbial communities based on culturing techniques detects only a small fraction of the microbial community [27], it has the advantage of having pure isolates than can be used in further studies. Using this approach, our study demonstrated that the microbial community of the studied eggshells was diverse and composed of a variety of heterotrophic bacteria. *Pseudomonas*, *Bacillus* and *Exiguobacterium* were the genera, *B*. *pumilus* and *E*. *undae* were the species most frequently found in both the cuckoo and great reed warbler eggshell samples. *P*. *fluorescens* and *P*. *putida* were more frequently detected on great reed warbler than on cuckoo eggshells.

Soler et al. [4] found with the analytical method of selective growth media that great spotted cuckoo eggshells harboured lower bacterial densities than those of their Eurasian magpie hosts in the same nests. In this study the parasitic eggs adapted better to environments with a high risk of bacterial contamination than did those of their magpie hosts, although this study identified larger groups of bacterial isolates from eggshells on only selective media KF, HE and VJ.

Previous studies showed that avian incubation decreased bacterial communities and reduced their growth on eggshells [10, 28, 29]. Although we did not study incubation effects in details, we showed that in the incubation stage cuckoo and great reed warbler eggs reached similar bacterial communities. We sequenced the 16S rRNA genes of 177 representative isolates and showed that bacterial community changed through brood parasitism, and cuckoo as a bacterial vector contaminated host eggs, and therefore influenced the hygienic condition of nests.

Some of the detected bacteria may influence egg viability [30, 31]. For example, *Pseudomonas* is a common bacterium in bird nests that could potentially be pathogenic [32–40]. We identified some species of the *Exiguobacterium* genus on avian eggshells at the first time, which are the members of the low GC phyla of *Firmicutes*. The species in the *Exiguobacterium* genus are globally diverse organisms that are found in a variety of environments, including microbialites. Collins et al. [41] described the genus *Exiguobacterium* with the characterization of *E*. *aurantiacum* strain DSM6208T from an alkaline potato processing plant. It has been found in areas covering a wide range of temperatures (minimum: -12°C; maximum: 55°C) including glaciers in Greenland and hot springs in the Yellowstone Park, Wyoming, and has been isolated from ancient permafrost in Siberia [42].

The nestlings of some avian brood parasitic species, like non-evictor cuckoos (e.g. the great spotted cuckoo) and cowbirds (*Molothrus spp*.) in America, cannot eliminate host eggs or hatchlings from the nest, so they often grow up together with hosts' own nestlings [14]. By contrast, hatchlings of the evictor brood parasites (e.g. the common cuckoo) evict all eggs or hatchlings from the nest [13, 26], and only one cuckoo chick can survive and grow up per parasitized nest, even if the clutch was parasitized by multiple cuckoo eggs [43]. Even if a cuckoo egg spends a short time in the nest before being ejected by its host (a typical antiparasitic defence mechanism [14]), it still has the opportunity to transfer new bacteria into the nest, onto the surface of the host eggs, or, indirectly, to the bill, skin or plumage of the incubating birds. Our study revealed that cuckoo eggs' bacterial community also became more similar to that of host eggs during incubation in contrast with their state in the laying period. However, the bacteria we found on cuckoo eggshells could be neutral for the hatchability of the eggs, and so we measured no difference in the hatching rates of host eggs in parasitized vs. non-parasitized nests. Future studies should clarify the exact mechanism of these mutual infections, as well as how these infections affect embryo health both in hosts and brood parasites. Although the eggs of the two bird species are similar in size (volume) in our study area [44, 45], the cuckoo eggshell is thicker [45–47] and contains more pores than that of great reed warblers [45]. As eggshell cuticles are important components of antimicrobial defence in wild birds, increased porosity might facilitate microbial infection, especially in a humid environment [48]. We also suggest future studies to compare how generalist and specialist brood parasites can overcome the problem of the high diversity of eggshell bacterial loads in different host species in a multihost situation (e.g. [49, 50]) vs. when there is only one main host, like in the present study.

## Supporting information

S1 Table Identification of the 177 bacterial strains isolated from the eggshells of common cuckoo and great reed warbler, based on BLAST analysis of the 16S rRNA gene sequences. (PDF)

S1 Fig Relative abundance of four bacterial phyla in samples. (TIF)

S2 Fig Maximum Likelihood tree of the eggshell bacterial isolates from parasitized nests. (TIF)
