# Supplementary material for: Common cuckoos (Cuculus canorus) affect the bacterial diversity of the eggshells of their great reed warbler (Acrocephalus arundinaceus) hosts
Source: PLoS One. 2018 Jan 19;13(1):e0191364. doi: 10.1371/journal.pone.0191364 (PMC5774785; doi:10.1371/journal.pone.0191364)
Supplement: S1 Table — (PDF) [file pone.0191364.s001.pdf]

Table S1 Identification of the 177 bacterial strains isolated from the eggshells of common cuckoo and great reed warbler, based on BLAST analysis of the 16S rRNA gene sequences

Nest ID	Representative isolate ID	Treatment	Type of medium	Colony color	Cell morphology	Gram stain	Nearest phylogenetic neighbor	16S rRNA gene sequence similarity in %	Accession	Phylum
11	55_5	pci	TSA	white	rod	-	Acinetobacter sp.	99%	JX137278.1	Proteobacteria
	55_1	pci	TSA	red	rod to cocci	+	Arthrobacter agilis	99%	FR682668.1	Proteobacteria
	52_1	pci	TSA	white	rod	+	Bacillus amyloliquefaciens	100%	KP334099.1	Firmicutes
	50_7	pci	TSA	white	rod	+	Bacillus subtilis	100%	CP011534.1	Firmicutes
	52_23	pgi	TSA	orange	ovoid or short rod	+	Exiguobacterium undae	100%	NR_043477.1	Firmicutes
	43_34	pgi	HE	green	rod	-	Pseudomonas fluorescens	100%	CP003041.1	Proteobacteria
	46_30	pgi	TSA	white	rod	-	Pseudomonas fluorescens	100%	CP003041.1	Proteobacteria
	49_1	pgi	TSA	white	rod	-	Pseudomonas fluorescens	100%	CP003041.1	Proteobacteria
20	50_1	ngn	TSA	white	rod	+	Bacillus pumilus	100%	KR088392.1	Firmicutes
	50_2	ngn	TSA	white	rod	+	Bacillus pumilus	100%	KR088392.1	Firmicutes
	51_31	ngn	TSA	orange	rod	-	Pseudomonas fluorescens	100%	CP005975.1	Proteobacteria
22	54_24	pci	TSA	orange	rod	+	Bacillus cereus	100%	KT354272.1	Firmicutes
	54_28	pci	TSA	orange	rod	-	Brevundimonas vesicularis	99%	AJ007801.1	Proteobacteria
	50_21	pci	TSA	white	rod	-	Pseudomonas reinekei	100%	NR_042541.1	Proteobacteria
	54_1	pgi	TSA	orange	rod	+	Bacillus pumilus	100%	KR088392.1	Firmicutes
	54_2	pgi	TSA	orange	rod	+	Bacillus pumilus	100%	KR088392.1	Firmicutes
39	49_9	pcn	TSA	white	rod	+	Bacillus pumilus	100%	KR088392.1	Firmicutes
	53_35	pgn	TSA	orange	ovoid or short rod	+	Exiguobacterium undae	100%	NR_043477.1	Firmicutes
40	40_3	pci	VJ	black	rod	-	Acinetobacter lwoffii	100%	KR054982.1	Proteobacteria
	40_4	pci	VJ	black	ovoid or short rod	+	Exiguobacterium undae	100%	NR_043477.1	Firmicutes
	48_1	pgi	TSA	white	rod	-	Acinetobacter johnsonii	99%	AB681724.1	Proteobacteria
	52_25	pgi	TSA	white	rod	-	Acinetobacter johnsonii	99%	LN774366.1	Proteobacteria
	48_23	pgi	TSA	white	rod	-	Pseudomonas lurida	100%	NR_042199.1	Proteobacteria
45	52_27	pgi	TSA	orange	ovoid or short rod	+	Exiguobacterium undae	100%	NR_043477.1	Firmicutes
47	48_31	ngn	TSA	white	rod	+	Bacillus pumilus	100%	KR088392.1	Firmicutes
	50_12	ngn	TSA	white	rod	+	Bacillus pumilus	100%	KR088392.1	Firmicutes
	50_13	ngn	TSA	white	rod	+	Bacillus pumilus	100%	KR088392.1	Firmicutes
	53_29	ngn	TSA	orange	rod	+	Bacillus thuringiensis	99%	AP014864.1	Firmicutes
51	49_14	ngi	TSA	white	rod	+	Bacillus pumilus	100%	KR088392.1	Firmicutes
52	47_11	ngn	TSA	white	rod	-	Acinetobacter johnsonii	100%	KC790286.1	Proteobacteria

Table S1 (continued)

Nest ID	Representative isolate ID	Treatment	Type of medium	Colony color	Cell morphology	Gram stain	Nearest phylogenetic neighbor	16S rRNA gene sequence similarity in %	Accession	Phylum
	51_22	ngn	TSA	orange	ovoid or short rod	+	Exiguobacterium undae	100%	AY444838.1	Firmicutes
	50_20	ngn	TSA	white	rod	-	Pseudomonas fluorescens	100%	KR054991.1	Proteobacteria
	44_1	ngn	HE	green	rod	-	Pseudomonas sp.	100%	KR088657.1	Proteobacteria
101	51_34	ngi	TSA	white	rod	+	Bacillus amyloliquefaciens	100%	KT833128.1	Firmicutes
209	46_34	pcn	TSA	white	rod	+	Bacillus pumilus	100%	KR088392.1	Firmicutes
	53_19	pcn	TSA	orange	ovoid or short rod	+	Exiguobacterium undae	100%	NR_043477.1	Firmicutes
	48_11	pcn	TSA	white-orange	rod	-	Stenotrophomonas rhizophila	100%	KR055001.1	Proteobacteria
	55_15	pgn	TSA	yellow-orange	rod	-	Chryseobacterium indoltheticum	100%	NR_042926.1	Bacteroidetes
	46_5	pgn	TSA	white	rod	-	Pseudomonas orientalis	100%	KC834315.1	Proteobacteria
	50_29	pgn	TSA	white	rod	-	Pseudomonas orientalis	100%	KC834315.1	Proteobacteria
	50_31	pgn	TSA	white	rod	-	Pseudomonas orientalis	100%	KC834315.1	Proteobacteria
	53_10	pgn	TSA	orange	rod	-	Stenotrophomonas rhizophila	100%	KR055001.1	Proteobacteria
210	47_12	ngn	TSA	white	rod	-	Acinetobacter calcoaceticus	100%	KR088359.1	Proteobacteria
	42_34	ngn	HE	green	rod	-	Pantoea agglomerans	99%	FJ357809.1	Proteobacteria
	42_31	ngn	HE	green	rod	-	Pseudomonas lurida	100%	NR_042199.1	Proteobacteria
	54_37	ngn	TSA	orange	rod	-	Pseudomonas putida	100%	KC195899.1	Proteobacteria
	48_14	ngn	TSA	white-brown	rod	-	Stenotrophomonas rhizophila	100%	KR055001.1	Proteobacteria
	53_37	ngn	TSA	orange	rod	-	Stenotrophomonas rhizophila	99%	KR055001.1	Proteobacteria
217	44_2	pgi	HE	green	rod	-	Acinetobacter calcoaceticus	100%	JQ781626.1	Proteobacteria
	47_2	pgi	TSA	white	rod	-	Acinetobacter calcoaceticus	100%	KR088359.1	Proteobacteria
	42_10	pgi	HE	green	rod	-	Erwinia rhapontici	99%	KR054974.1	Proteobacteria
	52_29	pgi	TSA	orange	ovoid or short rod	+	Exiguobacterium undae	100%	NR_043477.1	Firmicutes
	44_10	pgi	HE	green	rod	-	Pseudomonas orientalis	100%	KC834315.1	Proteobacteria
222	44_17	ngn	HE	green	rod	-	Pseudomonas fluorescens	99%	EU439419.1	Proteobacteria
	47_22	ngn	TSA	white	rod	-	Pseudomonas fluorescens	100%	NR_102835.1	Proteobacteria
224	46_36	pcn	TSA	white	cocci	+	Staphylococcus caprae	99%	KR632505.1	Firmicutes
	53_16	pgn	TSA	orange	rod	-	Pseudomonas fluorescens	100%	CP003041.1	Proteobacteria
232	51_24	pci	TSA	white	rod	-	Acinetobacter sp.	100%	KR088441.1	Proteobacteria
	55_17	pci	TSA	white	rod	-	Acinetobacter sp.	100%	KR088441.1	Proteobacteria
	55_3	pci	TSA	white	rod	-	Acinetobacter sp.	100%	KR088441.1	Proteobacteria

Table S1 (continued)

Nest ID	Representative isolate ID	Treatment	Type of medium	Colony color	Cell morphology	Gram stain	Nearest phylogenetic neighbor	16S rRNA gene sequence similarity in %	Accession	Phylum
	55_12_2	pci	TSA	pale yellow	rod	+	Arthrobacter nitroguajacolicus	100%	HF584979.1	Proteobacteria
	54_10	pci	TSA	orange	rod	+	Bacillus pumilus	100%	KR088392.1	Firmicutes
	54_8	pci	TSA	orange	rod	+	Bacillus pumilus	100%	KR088392.1	Firmicutes
	55_16	pgi	TSA	yellow-orange	rod	+	Exiguobacterium acetylicum	99%	NR_043479.1	Firmicutes
	42_37	pgi	HE	green	rod	-	Pseudomonas putida	100%	AP013070.1	Proteobacteria
	43_38	pgi	HE	green	rod	-	Pseudomonas putida	100%	AP013070.1	Proteobacteria
	47_7	pgi	TSA	white	rod	-	Pseudomonas putida	100%	AP013070.1	Proteobacteria
	49_3	pgi	TSA	white	rod	-	Pseudomonas putida	100%	AP013070.1	Proteobacteria
249	50_41	pci	TSA	white	rod	+	Bacillus pumilus	100%	KR088392.1	Firmicutes
	54_34	pci	TSA	orange	cocci	+	Kocuria rhizophila	99%	NR_074786.1	Actinobacteria
	50_34	pci	TSA	white	rod	-	Pseudomonas fluorescens	100%	CP005975.1	Proteobacteria
	46_29	pci	TSA	white	rod	-	Pseudomonas orientalis	100%	KC834315.1	Proteobacteria
	47_4	pgi	TSA	white	rod	+	Bacillus pumilus	100%	KR088392.1	Firmicutes
	52_12	pgi	TSA	orange	rod	+	Exiguobacterium acetylicum	99%	NR_043479.1	Firmicutes
509	52_2_1	pci	TSA	white	rod	-	Acinetobacter johnsonii	99%	LN774366.1	Proteobacteria
	47_40	pci	TSA	white	rod	-	Acinetobacter lwoffii	100%	KR054982.1	Proteobacteria
	42_24	pci	HE	green	rod	-	Enterobacter amnigenus	99%	HF562865.1	Proteobacteria
	42_25	pci	HE	green	rod	-	Enterobacter amnigenus	99%	HF562865.1	Proteobacteria
	42_6	pci	HE	green	rod	-	Enterobacter amnigenus	99%	DQ223879.1	Proteobacteria
	42_7	pci	HE	green	rod	-	Enterobacter amnigenus	99%	DQ223879.1	Proteobacteria
	44_13	pci	HE	green	rod	-	Enterobacter amnigenus	99%	HF562865.1	Proteobacteria
	40_24	pci	VJ	black	ovoid or short rod	+	Exiguobacterium undae	100%	NR_043477.1	Firmicutes
	44_14	pci	HE	green	rod	-	Lelliottia amnigena	99%	KR054963.1	Proteobacteria
	51_1	pci	TSA	white	rod	-	Pseudomonas fluorescens	100%	CP005975.1	Proteobacteria
	52_2_2	pci	TSA	pale orange	rod	-	Sphingobacterium faecium	99%	FJ950587.1	Bacteroidetes
	47_19	pgi	TSA	white	rod	-	Acinetobacter johnsonii	99%	AB681724.1	Proteobacteria
	40_42	pgi	VJ	black	rod	+	Bacillus subtilis	100%	CP011534.1	Firmicutes
	49_6	pgi	TSA	white	rod	-	Comamonas terrigena	99%	JF703644.1	Proteobacteria
	40_28	pgi	VJ	black	ovoid or short rod	+	Exiguobacterium undae	100%	NR_043477.1	Firmicutes
	49_5	pgi	TSA	white	rod	-	Pseudomonas fluorescens	100%	KR054992.1	Proteobacteria

Table S1 (continued)

Nest ID	Representative isolate ID	Treatment	Type of medium	Colony color	Cell morphology	Gram stain	Nearest phylogenetic neighbor	16S rRNA gene sequence similarity in %	Accession	Phylum
	43_30	pgi	HE	green	rod	-	Pseudomonas putida	99%	EU118779.1	Proteobacteria
520	44_8	pgi	HE	green	rod	-	Acinetobacter sp.	99%	JX899632.1	Proteobacteria
	47_27	pgi	TSA	white	rod	-	Acinetobacter sp.	100%	LN870383.1	Proteobacteria
	52_15	pgi	TSA	white	rod	+	Bacillus amyloliquefaciens	100%	KT833128.1	Firmicutes
	42_14	pgi	HE	green	rod	-	Pantoea anthophila	99%	NR_116113.1	Proteobacteria
	52_14	pgi	TSA	orange	rod	-	Pantoea anthophila	99%	NR_116113.1	Proteobacteria
	47_35	pgi	TSA	white	rod	-	Pseudomonas reinekei	100%	NR_042541.1	Proteobacteria
521	48_12	pcn	TSA	white	rod	-	Acinetobacter johnsonii	100%	KC790286.1	Proteobacteria
	40_18	pcn	VJ	black	ovoid or short rod	+	Exiguobacterium undae	100%	NR_043477.1	Firmicutes
	43_1	pcn	HE	green	rod	-	Pseudomonas putida	100%	FM211694.1	Proteobacteria
	40_8	pgn	VJ	black	rod	+	Bacillus subtilis	100%	CP011534.1	Firmicutes
	40_37	pgn	VJ	black	ovoid or short rod	+	Exiguobacterium undae	100%	NR_043477.1	Firmicutes
	48_27	pgn	TSA	white	rod	-	Pseudomonas fluorescens	100%	KR054992.1	Proteobacteria
	48_29	pgn	TSA	white	rod	-	Pseudomonas fluorescens	100%	KR054992.1	Proteobacteria
	40_7	pgn	VJ	black	rod	-	Pseudomonas putida	100%	KC195899.1	Proteobacteria
523	51_43	pcn	TSA	orange	ovoid or short rod	+	Exiguobacterium undae	100%	NR_043477.1	Firmicutes
	46_37	pcn	TSA	white	rod	-	Pseudomonas poae	100%	CP004045.1	Proteobacteria
	49_21	pcn	TSA	white	rod	-	Pseudomonas putida	100%	AP013070.1	Proteobacteria
	51_26	pcn	TSA	orange	rod	-	Pseudomonas rhizosphaerae	100%	LN774439.1	Proteobacteria
	51_16	pgn	TSA	white	rod	+	Bacillus pumilus	100%	KR088392.1	Firmicutes
	51_29	pgn	TSA	orange	rod	+	Bacillus thuringiensis	100%	AP014864.1	Firmicutes
526	51_37	pgn	TSA	white	rod	+	Bacillus amyloliquefaciens	100%	KT833128.1	Firmicutes
540	54_12	ngn	TSA	orange	cocci	+	Kocuria rhizophila	99%	NR_074786.1	Actinobacteria
	48_6	ngn	TSA	white	rod	-	Pseudomonas putida	100%	KC195899.1	Proteobacteria
	46_16	ngn	TSA	white	rod	-	Pseudomonas syringae	100%	CP005970.1	Proteobacteria
541	43_21	ngi	HE	green	rod	-	Pseudomonas putida	100%	FM211694.1	Proteobacteria
	52_4	ngi	TSA	orange	rod	-	Pseudomonas putida	100%	FM211694.1	Proteobacteria
542	50_5	pci	TSA	white	rod	+	Bacillus pumilus	100%	KR088392.1	Firmicutes
	52_6	pci	TSA	orange	rod	-	Pseudomonas putida	100%	FM211694.1	Proteobacteria
	52_16	pgi	TSA	orange	ovoid or short rod	+	Exiguobacterium undae	100%	NR_043477.1	Firmicutes

Table S1 (continued)

Nest ID	Representative isolate ID	Treatment	Type of medium	Colony color	Cell morphology	Gram stain	Nearest phylogenetic neighbor	16S rRNA gene sequence similarity in %	Accession	Phylum
	46_24	pgi	TSA	white	rod	+	Sporosarcina globispora	100%	AM237400.1	Firmicutes
545	51_18	ngn	TSA	white	rod	+	Bacillus amyloliquefaciens	100%	KT833128.1	Firmicutes
	53_31	ngn	TSA	orange	ovoid or short rod	+	Exiguobacterium undae	100%	NR_043477.1	Firmicutes
	54_14	ngn	TSA	orange	cocci	+	Kocuria rhizophila	99%	NR_074786.1	Actinobacteria
556	54_26	ngi	TSA	orange	ovoid or short rod	+	Exiguobacterium undae	100%	NR_043477.1	Firmicutes
557	49_13	pci	TSA	white	rod	+	Bacillus pumilus	100%	KR088392.1	Firmicutes
	48_24	pci	TSA	white	rod	-	Enterobacter aerogenes	99%	KF598982.1	Proteobacteria
	52_10	pci	TSA	orange	rod	+	Exiguobacterium acetylicum	99%	NR_043479.1	Firmicutes
	55_6	pci	TSA	yellow-orange	rod	+	Exiguobacterium acetylicum	99%	NR_043479.1	Firmicutes
	53_2	pgi	TSA	orange	rod	+	Bacillus pumilus	100%	KR088392.1	Firmicutes
	51_42	pgi	TSA	orange	ovoid or short rod	+	Exiguobacterium undae	100%	NR_043477.1	Firmicutes
	53_1	pgi	TSA	orange	rod	-	Pseudomonas fluorescens	100%	KR054991.1	Proteobacteria
	43_25	pgi	HE	green	rod	-	Pseudomonas putida	100%	EU118779.1	Proteobacteria
558	48_17	pcn	TSA	white	rod	-	Acinetobacter johnsonii	100%	KC790286.1	Proteobacteria
	53_25	pcn	TSA	orange	ovoid or short rod	+	Exiguobacterium undae	100%	NR_043477.1	Firmicutes
	51_33	pcn	TSA	orange	rod	-	Pantoea agglomerans	99%	FJ357809.1	Proteobacteria
	42_27	pgn	HE	green	rod	-	Acinetobacter johnsonii	99%	LN774366.1	Proteobacteria
	42_28	pgn	HE	green	rod	-	Acinetobacter johnsonii	99%	LN774366.1	Proteobacteria
	48_2	pgn	TSA	white	rod	-	Acinetobacter johnsonii	100%	AB681724.1	Proteobacteria
567	53_7	ngn	TSA	orange	rod	-	Pseudomonas fluorescens	100%	CP003041.1	Proteobacteria
	53_9	ngn	TSA	orange	rod	-	Pseudomonas putida	100%	AP013070.1	Proteobacteria
571	49_16	ngn	TSA	white	rod	+	Bacillus amyloliquefaciens	100%	KT833128.1	Firmicutes
	47_26	ngn	TSA	white	rod	+	Bacillus pumilus	100%	KR088392.1	Firmicutes
	51_5	ngn	TSA	white	rod	+	Bacillus pumilus	100%	KR088392.1	Firmicutes
	54_20	ngn	TSA	orange	cocci	+	Kocuria rhizophila	99%	NR_074786.1	Actinobacteria
575	48_25	ngn	TSA	white	rod	-	Acinetobacter lwoffii	100%	KR054982.1	Proteobacteria
	52_37	ngn	TSA	orange	ovoid or short rod	+	Exiguobacterium undae	100%	NR_043477.1	Firmicutes
	54_22	ngn	TSA	orange	cocci	+	Kocuria rhizophila	99%	NR_074786.1	Actinobacteria
606	40_17	ngi	VJ	black	ovoid or short rod	+	Exiguobacterium undae	100%	NR_043477.1	Firmicutes
	43_22	ngi	HE	green	rod	-	Pseudomonas corrugata	99%	HE586390.1	Proteobacteria

Table S1 (continued)

Nest ID	Representative isolate ID	Treatment	Type of medium	Colony color	Cell morphology	Gram stain	Nearest phylogenetic neighbor	16S rRNA gene sequence similarity in %	Accession	Phylum
	52_36	ngi	TSA	orange	rod	-	Pseudomonas putida	100%	AP013070.1	Proteobacteria
609	50_6	pci	TSA	white	rod	+	Bacillus amyloliquefaciens	100%	KT833128.1	Firmicutes
	51_19	pci	TSA	white	rod	+	Bacillus subtilis	100%	CP011534.1	Firmicutes
	53_14	pci	TSA	orange	ovoid or short rod	+	Exiguobacterium undae	100%	NR_043477.1	Firmicutes
	47_5	pgi	TSA	white	rod	+	Bacillus subtilis	100%	CP011534.1	Firmicutes
610	47_33	pci	TSA	white	rod	+	Bacillus pumilus	100%	KR088392.1	Firmicutes
	49_11	pci	TSA	white	rod	+	Bacillus pumilus	100%	KR055035.1	Firmicutes
	40_43	pci	VJ	black	ovoid or short rod	+	Exiguobacterium undae	100%	NR_043477.1	Firmicutes
	51_20	pci	TSA	translucent orang	rod	-	Pseudomonas graminis	100%	HE648152.1	Proteobacteria
	50_23	pci	TSA	dirty white	rod	-	Stenotrophomonas rhizophila	100%	KR055001.1	Proteobacteria
	52_33	pgi	TSA	white	rod	-	Acinetobacter lwoffii	100%	KR054982.1	Proteobacteria
	40_32	pgi	VJ	black	(coccoid) rod	+	Carnobacterium divergens	99%	AB598939.1	Firmicutes
	40_33	pgi	VJ	black	ovoid or short rod	+	Exiguobacterium undae	100%	NR_043477.1	Firmicutes
	52_34	pgi	TSA	orange	rod	-	Pseudomonas fluorescens	100%	CP003041.1	Proteobacteria
717	43_10	pcn	HE	green	rod	-	Erwinia persicina	99%	LM651373.1	Proteobacteria
	49_19	pcn	TSA	white	rod	-	Erwinia persicina	99%	LM651373.1	Proteobacteria
	40_1	pgn	VJ	translucent	rod	-	Acinetobacter lwoffii	100%	KR054982.1	Proteobacteria
	40_2	pgn	VJ	translucent	rod	-	Acinetobacter lwoffii	100%	KR054982.1	Proteobacteria
	48_4	pgn	TSA	white	rod	-	Acinetobacter lwoffii	100%	KR054982.1	Proteobacteria
	53_28	pgn	TSA	orange	rod	+	Bacillus pumilus	97%	KR088392.1	Firmicutes
	40_11	pgn	VJ	black	ovoid or short rod	+	Exiguobacterium undae	100%	NR_043477.1	Firmicutes
	40_9	pgn	VJ	black	ovoid or short rod	+	Exiguobacterium undae	100%	NR_043477.1	Firmicutes
	53_27	pgn	TSA	orange	rod	-	Stenotrophomonas rhizophila	100%	KR055001.1	Proteobacteria
723	50_10	pcn	TSA	white	rod	+	Bacillus pumilus	100%	KR088392.1	Firmicutes
	47_9	pgn	TSA	white	rod	+	Bacillus pumilus	100%	KR088392.1	Firmicutes
	51_14	pgn	TSA	white	rod	+	Bacillus pumilus	100%	KR088392.1	Firmicutes
730	49_7	pci	TSA	white	rod	-	Pseudomonas fluorescens	100%	KR054991.1	Proteobacteria

Type of media was used TSA— tryptic soy agar, HE— Hectoen Enteral agar, VJ— Vogel-Johnsson agar; Treatments was pcn— parasited nest, cuckoo egg, non-incubated; pci— parasited nest, cuckoo egg, incubated; pgn— parasited nest, great reed warbler egg, non-incubated; pgi— parasited nest, great reed warbler egg, incubated; ngn— non-parasited nest, great reed warbler egg, non-incubated; ngi— non-parasited nest, great reed warbler egg, incubated.
