# Supplementary figures and images for: Common cuckoos (Cuculus canorus) affect the bacterial diversity of the eggshells of their great reed warbler (Acrocephalus arundinaceus) hosts
Source: PLoS One. 2018 Jan 19;13(1):e0191364. doi: 10.1371/journal.pone.0191364 (PMC5774785; doi:10.1371/journal.pone.0191364)

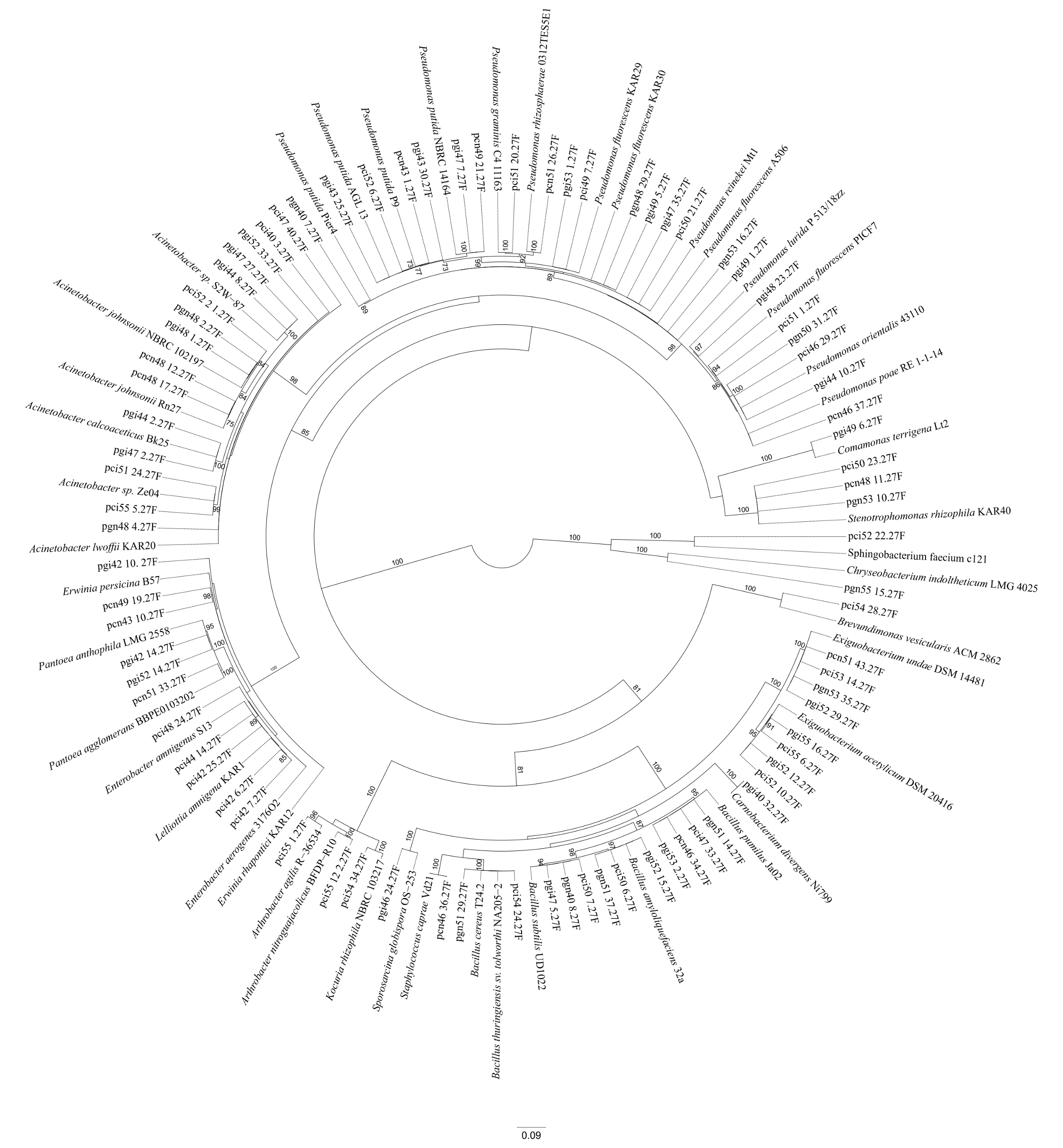

Supplement: S2 Fig — (TIF) [file pone.0191364.s003.tif]
